# The sculpting tool in bioprinting: research and application progress of sacrificial inks

**DOI:** 10.3389/fbioe.2025.1486459

**Published:** 2025-06-25

**Authors:** Li Jing, Hai Ci, Zihan Zhang, Zhenxing Wang, Bin Wang

**Affiliations:** ^1^ Department of Geryatrics, Union Hospital, Tongji Medical College, Huazhong University of Science and Technology, Wuhan, China; ^2^ Department of Plastic and Reconstructive Surgery, The First Affiliated Hospital of Shihezi University, Shihezi, China; ^3^ Department of Plastic and Reconstructive surgery, Union Hospital, Tongji Medical College, Huazhong University of Science and Technology, Wuhan, China; ^4^ Department of Plastic and Reconstructive Surgery, The Second People’s Hospital of China, Three Gorges University The Second People’s Hospital of Yichang, Hubei, China

**Keywords:** bioprinting, sacrificial inks, biomaterials, regenerative medicine, bioengineering

## Abstract

The core of bio-3D printing technology lies in the development and optimization of bio-inks. For a long time, researchers have been looking for bio-inks that can balance printability and cell function. However, traditional bio-inks often have limitations in meeting this balance, limiting the complexity and scale of printable structures. In recent years, the emergence of sacrificial inks has brought a major breakthrough in this field, allowing bio-inks that were originally not very suitable for printing to accurately construct larger and more complex structures. This ink is unique in that it is used to support and position the bio-ink but is removed after printing is complete, not as part of the final printed structure. The mild nature of the state transition and removal conditions allows for minimal damage to cell viability and print structure when the ink is “sacrificed.” This review will focus on the types of sacrificial inks and their two key applications in bioprinting: building intracranial vascular networks and improving bioink performance. We will summarize the current status, advantages, and challenges of these applications, aiming to provide readers with a comprehensive overview of the latest advances in the use of sacrificial inks in bioprinting. By sacrificing the application of ink, bioprinting technology can not only produce more realistic and complex tissue structures but also is expected to provide broader application prospects for clinical treatment and regenerative medicine in the future.

## 1 Introduction

Bio-3D printing technology, as a highly anticipated biomanufacturing technology in recent years, provides a powerful tool for constructing complex tissue structures with its high precision and controlled deposition capabilities in three-dimensional space (Szklanny et al., 2021). The core goal of this technology is to simulate the physiological structure and function of the human body to address the clinical issue of organ shortage (Skylar-Scott et al., 2019). Extrusion printing, stereolithography printing, inkjet printing, and laser-assisted printing have rapidly developed in the past decade, injecting new vitality into the field of bio-3D printing (Grigoryan et al., 2019; Miller et al., 2012; Schmidt and Belegratis, 2014; Zhao et al., 2022). In Table 1 we listed the process, advantages, limitations, applications of the four 3d bioprinting technologies.

**TABLE 1:** 3d bioprinting technologies.

	Process	Advantages	Limitations	Applications	References
Extrusion Printing	Pushing a semi-liquid^a^ material through a heated nozzle to deposit it layer by layer	Low cost, ease of use, material variety	Only applicable for viscous liquids, viscosities ranging from a minimum of 30 mPa·s to a maximum of 6 × 107 mPa·s. Lower Resolution, Slower speed	Widely used for prototyping, manufacturing final products, and creating customized parts	Agarwal et al. (2020), Kačarević et al. (2018), Prabhakaran et al. (2022)
Stereolithography (SLA) printing	Using a UV laser to cure and solidify a liquid photopolymer resin layer by layer	High precision, relatively fast speed, material efficiency	Damage to cells during photo curing, Post-Processing	Used in marking, coding, fine art, biosensors, and tissue engineering	Agarwal et al. (2020), Kačarević et al. (2018), Prabhakaran et al. (2022)
Inkjet printing	Spraying tiny droplets of ink onto a substrate to form the desired image or structure	Cost-effective, ability to print low viscosity biomaterials, fast speed	Poor functionality for vertical structures, low cell densities	Used in marking, coding, fine art, biosensors, and tissue engineering	Agarwal et al. (2020), Kačarević et al. (2018), Prabhakaran et al. (2022)
Laser-assisted printing	Using Laser-Induced Forward Transfer (LIFT), involves using a laser to transfer material from a donor substrate to a receiving substrate	Precision	Higher initial investment, Higher Energy Consumption	Extensively used in tissue engineering, drug discovery, and regenerative medicine	Agarwal et al. (2020), Kačarević et al. (2018), Prabhakaran et al. (2022)

^a^ Semi-liquid: substances that exhibit a dual-state behavior: they maintain structural rigidity at rest but flow like a liquid under applied shear forces (e.g., during extrusion).

However, achieving 3D printed scaffolds with both high morphological fidelity and excellent biological function remains a major technical challenge (Zhang et al., 2020). The choice of bio-ink plays a crucial role in obtaining scaffolds with both high morphological fidelity and biological function. Since bio-ink contains cellular components, the temperature, pressure, pH value, and mechanical properties such as stiffness and viscosity during the printing process must be carefully controlled to ensure cell viability and function are not compromised (Grigoryan et al., 2019; Ji and Guvendiren, 2017). Maintaining the balance between print performance and cell function is particularly critical in soft tissue fabrication. Harder, more viscous materials provide better shape fidelity but often have poorer biocompatibility, while softer, less viscous materials are more conducive to maintaining cell viability and function but typically have weaker extrusion and mechanical properties, making it difficult to ensure print stability and precision (Kang et al., 2016; Murphy and Atala, 2014). When these weaker mechanical bio-inks are used to print complex structures, collapse often occurs, preventing the formation of stable 3D structures.

In this context, the introduction of sacrificial inks has brought new breakthroughs to the field of bio-3D printing. Sacrificial inks, as special bio-inks, provide temporary support and positioning during the printing process and can be easily removed under specific conditions after printing, addressing the collapse problem in complex structure printing. Moreover, due to the mild and easy-to-implement removal process, they do not damage the surrounding cells and tissues, providing new ideas and methods for preparing 3D printed scaffolds with complex structures and excellent biological functions.

## 2 Requirements for sacrificial ink properties

Sacrificial ink plays a special role in 3D bioprinting, thus necessitating specific material properties. Firstly, since sacrificial ink is typically applied through extrusion printing, it must possess rheological characteristics suitable for this process (Xie et al., 2022). In Table 2 we discussed the four mechanical properties. This means that sacrificial ink should have the following attributes.

**TABLE 2:** Sacrificial ink properties.

Property	Principle	Significance	Problems
Appropriate viscosity	The resistance of a fluid to flow	Ensures smooth extrusion and layer formation	High viscosity can cause clogging; low viscosity can lead to poor shape retention
Yield stress	The stress at which a material begins to deform plastically	Prevents material from flowing under low stress, ensuring precise deposition	High yield stress can make extrusion difficult; low yield stress can cause sagging or spreading
Shear-thinning behavior	Viscosity decreases with increasing shear rate	Facilitates easy extrusion under pressure while maintaining shape post-extrusion	Excessive shear thinning can lead to instability and poor mechanical properties
Elastic recovery	The ability of a material to return to its original shape after deformation	Important for maintaining structural integrity and shape fidelity after printing	Poor elastic recovery can result in permanent deformation and loss of functionality

### 2.1 Appropriate viscosity

The sacrificial ink needs to have sufficiently high viscosity to prevent droplet formation during printing, thereby ensuring the precision and stability of the printed structures (Bakrani Balani et al., 2023; Barrulas and Corvo, 2023).

### 2.2 Yield stress

Before printing, the sacrificial ink should remain solid to maintain its shape and prevent flow. However, during extrusion, it must be able to flow smoothly through the print nozzle. This property, known as yield stress, ensures the stability of the ink when static and its flowability when dynamic (Mouser et al., 2016; Paxton et al., 2017).

### 2.3 Shear-thinning behavior

The sacrificial ink should exhibit shear-thinning properties, meaning its viscosity should decrease as the shear rate increases, facilitating easier passage through the print nozzle. This characteristic aids in achieving higher resolution and finer structures during printing (Naghieh et al., 2020; Petta et al., 2018).

### 2.4 Elastic recovery

While being extruded through the needle, the viscosity of the sacrificial ink should decrease to allow flow, but it should quickly recover its original viscosity upon exiting the needle to maintain the shape and stability of the printed structure. This self-healing behavior is crucial for printing complex structures (Highley et al., 2015; Olate-Moya et al., 2020).

In addition to these rheological requirements, sacrificial ink also needs an effective removal mechanism. This mechanism should allow the sacrificial material to separate from the printed structure without compromising its integrity. Common removal methods include dissolution in water, physical extraction, sol-gel transitions induced by temperature changes, and dissolution with chelating agents (Compaan et al., 2017; Kolesky et al., 2016; Mahdi et al., 2016; Mohanty et al., 2016).

## 3 Overview of common sacrificial inks

### 3.1 Gelatin

Gelatin, a partially hydrolyzed product of collagen, is a natural polymer material widely used in the biomedical field. Its unique temperature sensitivity makes it an ideal candidate for sacrificial inks. Gelatin can dissolve in water at higher temperatures (such as 37°C or higher) to form a solution and undergo gelation when the temperature decreases, forming a solid gel structure (Kang et al., 2016; Yang et al., 2023).

The principle of this thermoreversible gelation behavior lies in the interactions between gelatin molecular chains. As the temperature decreases, the thermal energy between molecules reduces, and van der Waals forces promote the formation of physical cross-linking points between molecular chains, constructing a uniform network structure. This structure restricts molecular mobility, giving gelatin gels good mechanical strength and shape stability (Xie et al., 2022). Gelatin’s mechanical strength varies significantly depending on its processing methods, a study observed that for neat gels, the storage modulus ranged between 9 and 13 kPa, with the storage modulus (G′) being one order of magnitude higher than the loss modulus (G″), highlighting their predominantly elastic behavior.

As a sacrificial ink, the significant advantages of gelatin are its excellent biocompatibility and biodegradability. Gelatin is non-toxic and non-irritating to cells and can rapidly degrade into natural amino acids in the body, being absorbed and utilized by the organism. Even if there is a small amount of gelatin residue during the removal process, it does not adversely affect the surrounding tissues and cells. These characteristics make gelatin a highly potential sacrificial ink material with broad application prospects in the field of bio-3D printing (Murphy and Atala, 2014; Ouyang et al., 2020; Shao et al., 2020).

The disadvantage of gelatin is low viscosity and unstable gelation. As a hydrolyzed derivative of collagen, it forms randomized macromolecular chains with heterogeneous structures. This irregularity reduces its ability to maintain consistent flow resistance (viscosity), especially in solutions at physiological temperatures (e.g., 37°C), gelatin’s gelation kinetics are influenced by factors like pH and ion concentration, which can lead to inconsistent crosslinking and mechanical weakness. This results in poor printability, low mechanical strength, and weak shape fidelity, limiting the use of gelatin for manufacturing complex structures. The printability window of gelatin-based bioinks is very narrow. Printability window refers to the range of process parameters (e.g., pressure, temperature, speed, voltage) within which a bioink can be successfully extruded and stabilized to form high-fidelity structures during bioprinting. A “narrow” printability window means the bioink is highly sensitive to parameter variations, requiring precise control to avoid printing failures (e.g., fiber breakage, structural collapse, or nozzle clogging). Introducing a polymer that can independently cross-link or cross-link with gelatin chains after printing can achieve higher printability and shape fidelity (Yang et al., 2023).

### 3.2 Pluronic F127

Pluronics, as amphiphilic triblock copolymers, play an important role in drug formulation and tissue engineering. Its structure consists of hydrophilic polyethylene glycol (PEG) and hydrophobic polypropylene glycol (PPO) alternately, forming a PEG-PPO-PEG triblock structure. This unique chemical structure gives Pluronic F127 temperature sensitivity, and its gelation behavior is closely related to concentration (Hopkins and de Bruyn, 2019).

In bio-3D printing, Pluronic F127 is used as a sacrificial ink due to its temperature sensitivity and sol-gel transition properties. At 20% (w/v) concentration, gels near 25°C–30°C (close to body temperature, ideal for biomedical applications). Lower concentrations (e.g., 15%) require higher temperatures (∼30°C–35°C) for gelation. At 20% (w/v), Storage Modulus ≈ 1,000–5,000 Pa (temperature-dependent; stronger at higher concentrations or temperatures near gelation). When the solution temperature is above its gelation temperature, high concentrations of Pluronic F127 can form a stable hydrogel, providing support for the printed structure. As the temperature decreases, it can achieve a reversible transition from gel to sol, making it easy to remove Pluronic F127 under mild conditions without damaging surrounding cells or tissues (Hou et al., 2023).

Although Pluronic F127 has significant advantages as a sacrificial ink in bio-3D printing, such as easy printing and removal, it also has some limitations. For example, its mechanical strength is low, stability is poor, rapid degradation, and relatively slow gelation process, which to some extent limits its wide use in complex tissue engineering applications (Akash and Rehman, 2015; Hopkins and de Bruyn, 2019; Khaliq et al., 2023; Singla et al., 2022).

### 3.3 Alginate

Alginate, especially sodium alginate, is a polysaccharide extracted from natural brown algae, receiving widespread attention in the biomedical field for its excellent biocompatibility. When sodium alginate comes into contact with calcium ions, an ion exchange reaction rapidly occurs, forming stable calcium alginate gels (Li et al., 2023). This gelation process is fast and reversible, making alginate an ideal candidate for sacrificial ink materials (Besiri et al., 2023; Murujew et al., 2021; Shan et al., 2024).

In bio-3D printing, the sacrificial function of alginate is primarily achieved through calcium chelating agents. These chelating agents can bind to calcium ions in calcium alginate gels, breaking the gel structure and liquefying it into a solution. This removal process is mild and effective, having minimal impact on surrounding cells and tissues (Li et al., 2021; Saeki et al., 2020).

However, despite the excellent biocompatibility and adjustable mechanical properties of alginate, there are still some challenges as sacrificial ink. For example, it is necessary to precisely control the concentration and distribution of calcium ions to achieve ideal gelation effects while avoiding adverse effects on cells (Mahdi et al., 2016; Wan et al., 2008).

### 3.4 Agarose

Agarose, a galactose polymer polysaccharide extracted from algae, occupies a place in the biomedical field for its unique thermosensitivity and thermoreversibility. The degree of hydroxyethylation affects its sol temperature, making agarose a potential sacrificial ink in bio-3D printing (Ren et al., 2022).

However, a significant problem with agarose as a sacrificial ink is that its removal process usually requires high-temperature conditions, posing a severe threat to cell viability. Typically melts between 85°C and 95°C. Higher concentrations (e.g., 2%–3%) require temperatures closer to 95°C. However, studies have shown that when the agarose mold is cast around a photo-crosslinked hydrogel through acrylic groups, agarose fibers can be easily removed through vacuum suction or manual means without damaging the overall structure. This success may be attributed to the lack of covalent chemical bonds between agarose chains and acrylic groups, reducing material adhesion (Wenger et al., 2022).

Although this method provides new possibilities for using agarose as sacrificial ink, it still has certain limitations. For example, the vascular network structure created using this method must be open, and during the removal process, mechanical stress may be exerted on adjacent bio-inks, compromising the overall structural integrity. Therefore, careful consideration of its removal mechanism and potential impact on the printed structure is required when applying agarose to bio-3D printing.

### 3.5 Polyvinyl alcohol (PVA)

Polyvinyl alcohol (PVA) is a synthetic polymer widely used in the biomedical field (Goh and Hashimoto, 2018). It has good biocompatibility, high water content, and high elasticity. The formation of PVA hydrogels mainly relies on weak non-covalent bonds such as hydrogen bonds and van der Waals forces between molecular chains, making it a potential candidate for sacrificial ink (Masri et al., 2023).

Compared to some natural hydrogels, PVA scaffolds printed through melt deposition have superior mechanical properties. Additionally, PVA is easily soluble in water or phosphate-buffered saline (PBS), allowing it to be removed from printed structures through a simple soaking process without using complex solvents (Khati et al., 2022; Shimizu et al., 2020; Shimizu et al., 2020).

Despite the many advantages of PVA as sacrificial ink, its removal process is relatively slow, highly hydrolyzed PVA requires a high dissolution temperature (∼100°C) and about 30 min, while the solubility of lower hydrolysis grades is very poor (Teodorescu et al., 2019). The longer soaking process may affect the structural stability of the overall printed scaffold. Therefore, considering its slow removal speed and potential impact on cell survival, its application in bio-3D printing must be carefully evaluated. In Table 3 we described the characteristics of common sacrificial inks.

**TABLE 3:** Characteristics of common sacrificial ink**s**.

	Advantage	Limitation	Removal strategies	Application	References
Gelatin	Unique temperature sensitivity, excellent biocompatibility and biodegradability, non-toxic and non-irritating	Low viscosity and unstable gelation, poor printability, low mechanical strength, and weak shape fidelity, limited for manufacturing complex structures	Elevated temperatures (37°C)	3D bioprinting of mesoscale pore networks	Shao et al. (2020)
Pluronic F127	Reversible transition from gel to sol, easy to remove under mild conditions without damaging surrounding cells or tissues	Low mechanical strength, poor stability, rapid degradation, and relatively slow gelation process	Lowered temperatures (4°C)	bioactive GelMA^a^ as extracellular matrix (ECM), Human Umbilical Vein Endothelial Cells (HUVECs) as cells to build vascularized tissue constructs	Kolesky et al. (2014)
Alginate	Excellent biocompatibility and adjustable mechanical properties	Need for precise control the concentration and distribution of calcium ions	Chelating agent	sodium alginate with silk fibroin as ECM,NIH 3T3 fibroblasts as cells to build 3D Silk Fibroin Cellular Constructs	Compaan et al. (2017)
Agarose	Easily removed through vacuum suction or manual means without damaging the overall structure	Overall structural integrity may be damaged during the removing process	Physical extraction	build 3D perfusable channel creation for biomedical applications	Ren et al. (2022)
Polyvinyl Alcohol (PVA)	Good biocompatibility, high water content, and high elasticity	Slow removal speed and potential impact on cell survival	Dissolution in aqueous solution	sodium alginate, agarose, and platelet-rich plasma (PRP) composite hydrogel as ECM, H9c2 cardiomyocytes and HUVECs as cells to bulid a bioengineered heart	Zou et al. (2020)

^a^ GelMA: a bioactive hydrogel precursor derived from gelatin through methacrylation, enabling photocrosslinking under ultraviolet or visible light.

## 4 Methods of printing with sacrificial ink

Based on the sequence and method of printing sacrificial ink and bioink, there are three main methods: supportive bath printing, sacrificial mold printing, and multi-material printing. In Table 4 we discussed the characteristics and differences of three printing methods.

**TABLE 4:** characteristics and differences of three printing methods.

	Sacrificial mold printing	Multi-material printing	Support bath printing
Process	Uses sacrificial materials to create molds that are later removed	Integrates different materials into a single print using various techniques	Extrudes liquid ink materials into a fluid bath to form 3D configurations
Materials	Typically involves biodegradable or easily removable materials	Combines materials with different properties (e.g., polymers, hydrogels)	Utilizes a fluid bath to support the printed structure
Complexity	Enables the creation of complex internal structures	Allows for complex geometries and functional materials	Provides *in situ* support for complex structures
Advantages	High precision in creating intricate structures. Biocompatibility	Versatility in material properties. Enhanced functionality	Reduces dependence on ink material’s cross-linkability. Broadens material selection
Disadvantages	Removal of sacrificial material can be challenging	Complexity in material integration. Potential for material incompatibility	Requires careful control of fluid bath properties

### 4.1 Supportive bath printing

Supportive bath printing is a technique where sacrificial ink is used to create supportive bath structures within a supporting bath. In this method, sacrificial ink is used to print the desired vascular channels or voids, with the surrounding support bath providing necessary structural support. After printing, the sacrificial ink is removed through an appropriate removal mechanism, leaving behind the required cavities or channels (Brunel et al., 2022).

A typical application of supportive bath printing is using Pluronic F127 as sacrificial ink. Pluronic F127 is a thermosensitive hydrogel with good biocompatibility and temperature responsiveness. It is liquid at low temperatures (e.g., 4°C) and can serve as a support bath. At higher temperatures (e.g., 37°C), it solidifies and can be used as sacrificial ink for printing. After solidifying the support bath through methods like photopolymerization or chemical cross-linking, the temperature is lowered to liquefy Pluronic F127, which is then expelled from the structure, forming the desired channels or cavities (Wu et al., 2011).

**FIGURE 1:**
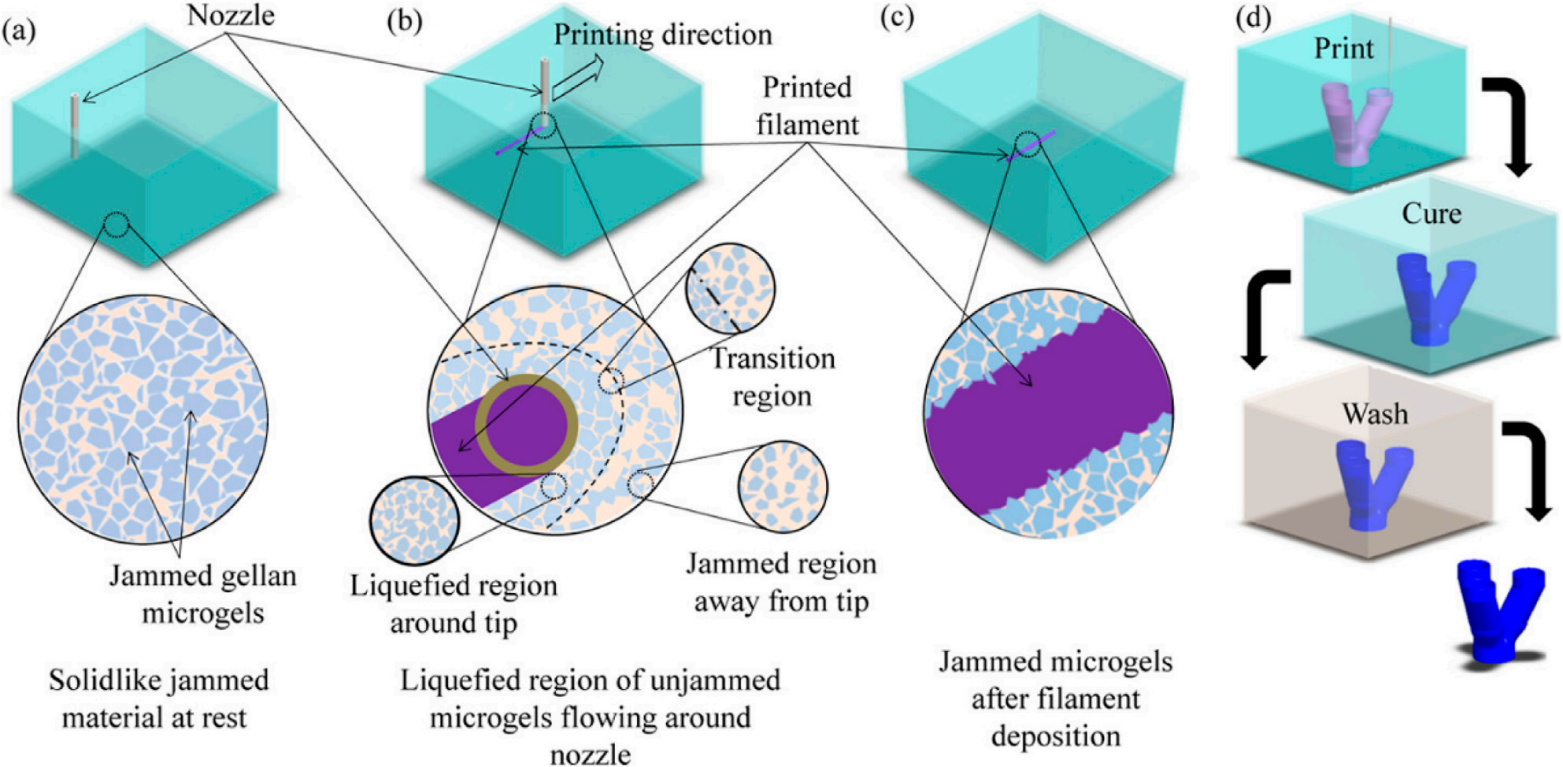
Support bath printing. Printing process schematics of print bath: **(a)** before printing, **(b)** during filament deposition showing local liquefaction, and **(c)** after filament deposition showing entrapped filament as well as **(d)** overall printing and post-processing steps to fabricate a branching tubular construct. Reprinted with permission from ref (Compaan et al., 2019). Copyright 2019 American Chemical Society.

Besides Pluronic F127, gelatin is another commonly used sacrificial ink in supportive bath printing. Gelatin is a natural polymer with good biocompatibility and degradability. It remains solid at low temperatures for printing but liquefies at near physiological temperatures (e.g., 37°C) and can be expelled from the structure. Using gelatin as sacrificial ink for supportive bath printing can create high cell density, vascularized, functional thick tissue structures, providing strong support for tissue engineering and regenerative medicine (Hua et al., 2021) (Figure 1).

### 4.2 Sacrificial mold printing

Sacrificial mold printing is another important application of sacrificial ink, especially when non-hydrogel materials with stringent requirements need to be manufactured. In this method, sacrificial ink is used to print a rigid 3D mold, which is subsequently used as a support structure for manufacturing the final product. An early example is the 2012 study by Miller et al., where they used thermal extrusion printing technology to print carbohydrate glass materials (including glucose, sucrose, and dextran) into rigid 3D grid structures (Miller et al., 2012). These structures solidified at room temperature and were then encapsulated by various cell-laden hydrogel materials. Once the hydrogel cross-linked, the carbohydrate grid, acting as sacrificial material, was easily dissolved in water or cell culture medium, forming hollow network channels. A limitation of this method is that, since carbohydrates are printed without other support structures, the resulting channel patterns are relatively simple and cannot mimic the complex vascular networks in the human body.

**FIGURE 2:**
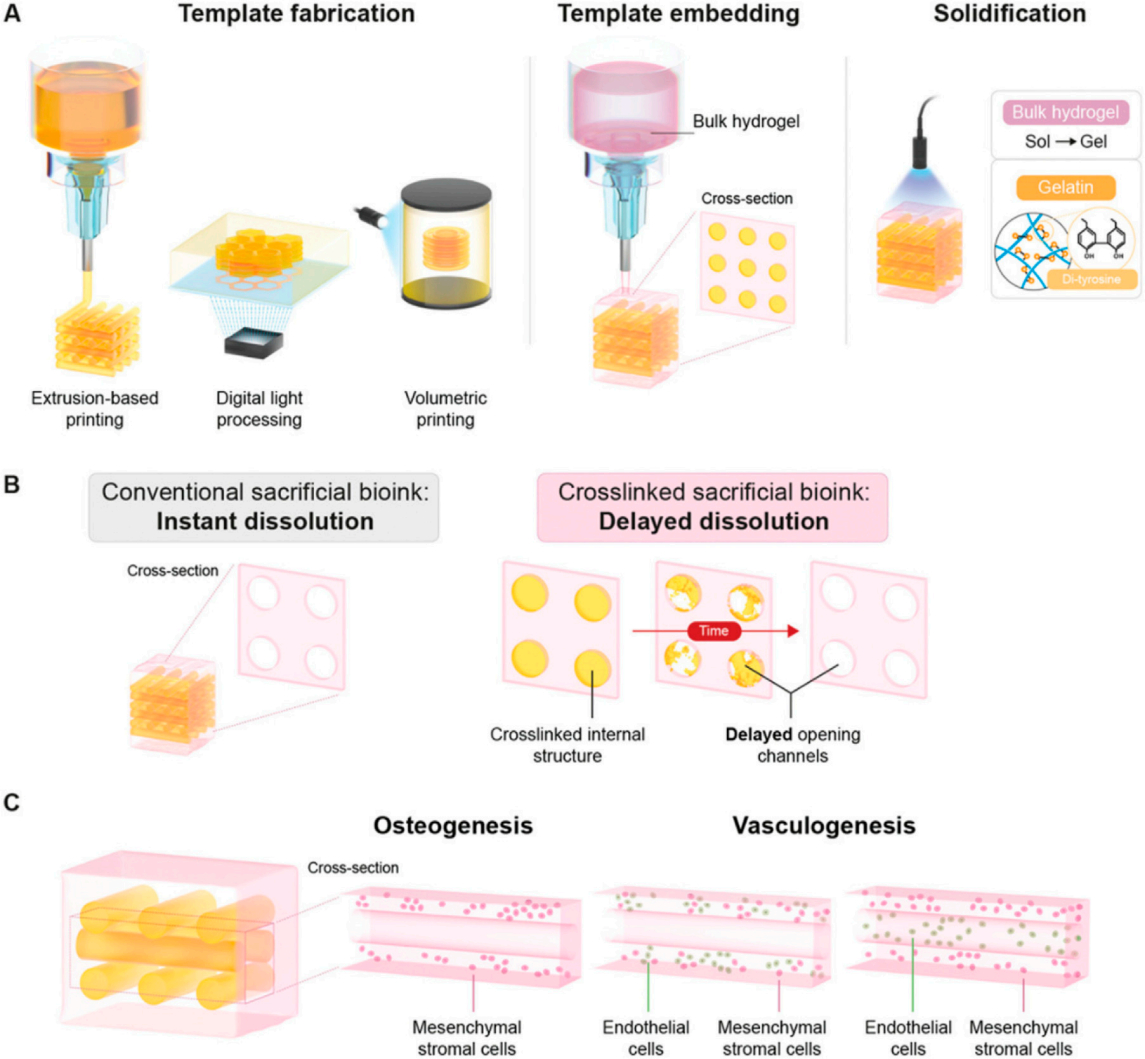
Sacrificial mold printing. Schematic overview of delayed dissolution sacrificial printing platform for temporal introduction of microchannels. **(A)** Fabrication of delayed dissolution sacrificial bioink consisting of pristine gelatin and the Ru/SPS photo-initiating system through extrusion-based printing (EBP), digital light processing (DLP), and volumetric bioprinting (VBP). After embedding of the sacrificial template in bulk hydrogel-precursor solution, the bulk hydrogel-precursor solution and gelatin sacrificial template is exposed to light to initiate photo-cross-linking. **(B)** Where conventional sacrificial templates dissolve rapidly and without temporal control, leaving open channels, the photo-cross-linked sacrificial gelatin templates demonstrate delayed dissolution, only leaving open channels over time and in a controllable manner. **(C)** The effect of the timing of sacrificial template dissolution on the behavior and functionality of encapsulated cells was assessed via osteogenesis and vasculogensis tissue models. Reproduced with permission from ref (Bram and Soliman, 2023); CC BY-NC-ND 4.0. Copyright^©^ 2023 by the authors.

To overcome this limitation, Miller et al. improved their approach in subsequent studies. They used laser sintering technology to print sugar materials (isomalt and corn starch) into complex branching scaffolds. The scaffold was then cast with hydrogel materials, and after the outer shell materials like Polydimethylsiloxane (PDMS), Polycaprolactone (PCL), Poly (ethylene glycol) diacrylate (PEGDA), agarose, silk fibroin, and fibrin cross-linked, the scaffold was immersed in water or PBS solution to dissolve (Kinstlinger et al., 2020). This method demonstrated that the vascular channels formed after removing the sacrificial ink had good connectivity, and endothelial cells infused into the lumens formed complex, dendritic vascular networks. This research showcased the potential of sacrificial mold printing for creating tissue engineering constructs with complex internal structures (Brassard et al., 2021) (Figure 2).

### 4.3 Multi-material printing

Multi-material printing technology, particularly in the field of bioprinting, has shown tremendous potential and application value. This technology allows the use of multiple different bioinks within the same printing process, each with its unique properties and functions, enabling the creation of more complex and biomimetic tissue structures (Grigoryan et al., 2019; Lee et al., 2019).

Gelatin, as a hydrogel sacrificial ink, plays an important role in multi-material printing. Due to its good biocompatibility and temperature sensitivity, gelatin is widely used to manufacture tissue structures with perfusable and branched pre-vascular networks. For example, research has demonstrated a method to fabricate centimeter-scale soft vascular tissues using multi-material bioprinting. They used a customized multi-stage temperature-controlled printer, loading GelMA-fibrin (GF) blend containing HUVEC bioink and gelatin sacrificial ink through two separate printheads (Lu et al., 2023). After printing, the sacrificial ink was removed to construct a 3D structure with stereoscopic branched vessels. *In vitro* perfusion culture showed that the loaded cells proliferated well, making it possible to construct complex tissues like the liver *in vitro*.

Researches further demonstrated the application of multi-material printing in creating more complex structures and functional tissues. They used temperature-sensitive gelatin as sacrificial ink and photocrosslinkable GelMA as bioink for synchronous printing. This printing method utilized gelatin’s support during the printing process, making the printed gel structure more stable. By incubating the printed structure at 37°C, the gelatin dissolved to form a continuous channel network. Additionally, they loaded HUVECs into the gelatin sacrificial ink, and as the gelatin liquefied, endothelial cells adhered and proliferated within the channels, achieving *in situ* endothelialization. This method addressed issues of uneven and uncontrollable cell seeding, providing new ideas for creating tissues with complex vascular networks (Szklanny et al., 2021).

**FIGURE 3:**
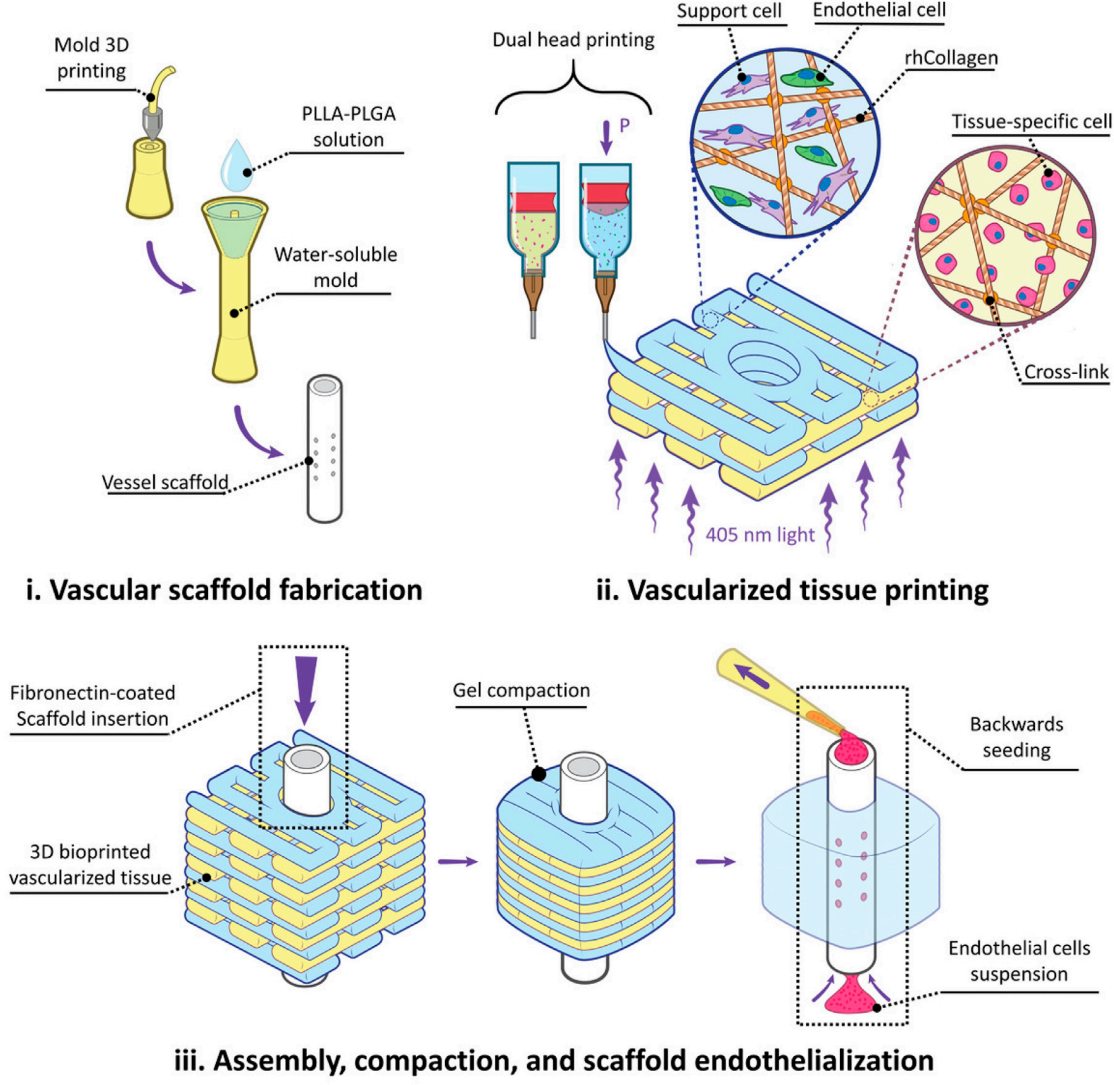
Multi-Material Printing. Figure Legend: Experiment flow for fabricating and implanting perfusable vascularized tissues. i) Water-soluble molds are 3D printed, filled with a Poly - L - lactic acid - Poly (lactic - co - glycolic acid) (PLLA-PLGA) polymer solution, lyophilized, and washed away, resulting in tubular fenestrated scaffolds (VascFold). ii) Recombinant human collagen methacrylate (rhCollMA) is used as a bioink to bioprint vascularized tissues. A dual head extrusion system is used to fabricate intercalated rhCollMA layers containing support and endothelial cells or tissue-specific cells. iii) Immediately after printing, a fibronectin-coated VascFold is inserted into the printed tissue channel and cultured for 2 days. The cells in the rhCollMA start organizing into functional tissues, exerting forces that compact the gel. The compaction stabilizes the printed rhCollMA around the scaffold and covers its side fenestrations. Then, endothelial cells are seeded into the VascFold lumen by applying negative pressure. Reproduced with permission from ref (Szklanny et al., 2021); CC BY-NC-ND 4.0. Copyright ^©^ 2021 by the authors.

Overall, the development of multi-material printing technology brings more possibilities and opportunities to the field of bioprinting. By combining different bioinks and sacrificial inks, we can create more biomimetic and complex tissue structures, better simulating the physiological environment within the human body. This is of great significance for the development of tissue engineering and regenerative medicine (Figure 3).

## 5 Challenges and prospects of sacrificial inks

Although sacrificial inks have brought new breakthroughs to the field of bio-3D printing, there are still some challenges to address in their application. For example, the mild and efficient removal process of sacrificial inks must be ensured without affecting the printed structure and cell function (Wan et al., 2008). Additionally, the properties and printability of sacrificial inks need further optimization to meet the requirements of various printing applications (Seymour et al., 2021).

Despite these challenges, the prospects for sacrificial inks in bio-3D printing are promising. With continuous technological innovation and in-depth research, sacrificial inks are expected to play a more critical role in tissue engineering and regenerative medicine, providing new solutions for the clinical treatment of complex diseases and the preparation of complex tissue structures (Cheng et al., 2023; Ji et al., 2019; Shao et al., 2020).
